# Understanding registered nurses’ career choices in home care services: a qualitative study

**DOI:** 10.1186/s12913-023-09259-0

**Published:** 2023-03-21

**Authors:** Guro Hognestad Haaland, Olaug Øygarden, Marianne Storm, Aslaug Mikkelsen

**Affiliations:** 1 Stavanger University Hospital, Stavanger, Norway; 2 Business School, University of Stavanger, Stavanger, Norway; 3 NORCE Norwegian Research Centre AS, Stavanger, Norway; 4 Department of Public Health, Faculty of Health Sciences, University of Stavanger, Stavanger, Norway; 5 Faculty of Health Sciences and Social Care, Molde University College, Molde, Norway

**Keywords:** Nursing, Career choices, Career events, Home care services, Sustainable workforce, HR policies, Management

## Abstract

**Background:**

The anticipated growth in number of older people with long-term health problems is associated with a greater need for registered nurses. Home care services needs enough nurses that can deliver high quality services in patients’ homes. This article improves our understanding of nurses’ career choices in home care services.

**Methods:**

A qualitative study using individual semi-structured interviews with 20 registered nurses working in home care services. The interviews were audio-recorded, transcribed and thematically analyzed.

**Results:**

The analysis resulted in three themes emphasizing the importance of multiple stakeholders and contextual factors, fit with nurses’ private life, and meaning of work. The results offer important insights that can be used to improve organizational policy and HR practices to sustain a workforce of registered nurses in home care services.

**Conclusion:**

The results illustrate the importance of having a whole life perspective to understand nurses’ career choices, and how nurses’ career preferences changes over time.

## Background

The anticipated growth in number of older people and earlier hospital discharge of patients with more complicated medical diagnosis is associated with a greater need for health care services at home [1–3]. Similarly to other Nordic countries, Norwegian municipalities are responsible for providing primary health care services in patients’ homes [4] irrespective of gender, age, geographical location or socioeconomic status [5]. Primary care services include home care services, nursing homes, municipal emergency care units, intermediate care, the provision of GPs and preventive services [4]. Consistent with previous research [6–9], this paper makes use of the term home care nurses referring to registered nurses who work in home care services. Home care services include nursing care and other forms of health care such as physiotherapy, occupational therapy or rehabilitation for either a short or a long period [5]. Health care delivered at home has become more complex, and registered nurses play a critical role providing care to sicker patients needing advanced care [8, 10]. The number of recipients receiving nursing care in their own homes has grown rapidly in the recent years [11], and the growth is expected to increase significantly also in the years to come [12]. With the worldwide shortage of nurses [13], there is an urgent need to understand what influences registered nurses’ career choices in home care services. This may provide information to ensure that registered nurses consider home care nursing as an attractive workplace for a lifelong career.

Traditionally, the choice of an occupation was associated with a linear career path and a secure employment within one organization. Nowadays, careers can be unpredictable and complex, and employees are not bound to their initial occupation [14]. In Norway, one in five registered nurses leave health care services ten years after graduating [15]. High turnover among nurses who leave their clinical jobs or profession is costly, because it is expensive to train and replace experienced nurses. The result is understaffing, which is a potential risk to patient safety [16]. Previous studies have mainly focused on nurse students’ career preferences, and identified that primary health care is not the preferred workplace for nursing students [17–19]; however, the likelihood of working in the municipal health and care services increases with time [20–22]. In a quantitative longitudinal study, Abrahamsen [23] identified how nurse students’ career expectations relate to their career choices. One year after graduation, choosing to work in nursing homes and home care nursing related to nurses’ expectations of achieving a management position. Ten years later, nurses’ choice to work in nursing homes and home care services rather related to nurses’ expectations to work part time, illustrating that the motives behind career choices change with time. Abrahamsen tested three dimensions of career expectations and emphasized the importance of additional knowledge of registered nurses career choices to improve recruitment and retention strategies in the less popular nursing fields such as home care services.

As contemporary careers are increasingly dynamic and complex, employees can make several career choices over time and adjust to external influences [24]. Nurses can change occupation, work in different organizations, have a permanent or temporary position, and apply for temporary unpaid leave to raise their children. The shortage of registered nurses means that they can choose between many career options. Researchers have investigated the most and least satisfying aspects of work in primary health care [25], nurses’ job satisfaction and quality of life [26, 27], and why home care nurses remain in their jobs [28, 29]. Results of these studies identified autonomy, work-life balance, interaction with patients, role diversity, and patient-family interaction as satisfying aspects of work in primary health care and influence nurses’ intention to remain. In contrast, low pay, lack of a career path, time constraints and workload have been identified as the least satisfying aspects of work in primary health care [25, 27]. Although these studies increased our knowledge of important aspects of nurses’ career in primary health care, there is still a need for a more detailed understanding of what affects nurses’ career choices in home care services [30, 31]. Since 2015, the municipalities have experienced an increase in challenges recruiting registered nurses to nursing homes and home care services [32], and previous studies have reported a lack of registered nurses with sufficient competence in primary health care services [33, 34]. This stresses the importance to increase our understanding of what influences registered nurses career choices in home care services. This paper seeks to address this gap by asking the following research question; *how do contextual and individual factors influence registered nurses’ career choices in home care services*? The results can provide home care services and other health care organizations with important information on how to provide human resource management practices and organizational policies in order to recruit, develop and retain registered nurses. Changing needs and motivations, and contextual demands affect person-career fit and people’s career choices over time [35]. Examples of registered nurses’ career choices are starting in home care services, working part-or full-time, changing hours of work, becoming a resource nurse, taking a specialization, or leaving home care services. We will use the sustainable career framework that provides a whole-life perspective on careers that is useful for understanding nurses’ career choices [24].

## Sustainable careers

Careers are dynamic, made up of choices and events over time that will determine their sustainability [24]. Unlike other career paradigms, the sustainable career perspective stresses the importance of context and the role of multiple stakeholders on the sustainability of employees’ career over time [36]. Sustainable careers draws on theories like selection optimization and compensation [37], conservation of resources [38] and self-determination theory [39], and emphasize the importance of resources and fulfillment of the psychological needs for autonomy, competence and relatedness for ensuring sustainable growth and continuity in one’s career [36]. Findings suggests that basic psychological needs relate to registered nurses’ turnover intention [40] and career commitment [41]. Something that is *sustainable* can last for a long time without being depleted or destroyed [24]. For nurses to have a long career, the home care services needs to create work conditions that endorse motivation and well-being, and nurses themselves needs to stay employable. Sustainable careers are characterized by happy, healthy and productive workers, and are defined as “sequences of career experiences reflected through a variety of patterns of continuity over time, thereby crossing several social spaces characterized by individual agency, herewith providing meaning to the individual” (24, p.7). In order to attract, motivate, develop and retain registered nurses over time, home care services should foster sustainable careers as unsustainable careers increase the risk for career turnover [42]. Three dimensions can be used to study sustainable careers, the person, the context and time [36].

The *person* dimension relates to agency and meaning [24]. *Agency* refers to making career choices that are consistent with individual’s needs and aspirations, or adapting to external changes and events [36]. To have a sustainable career over time, employees need to craft their career, which refers to “proactive behaviours […] to self-manage their career and that are aimed at attaining optimal person-career fit” [43, p. 175–176]. *Meaning* refers to people being mindful about what and who is important to them in their career, and this might change over time [36]. Meaning of work is associated with registered nurses’ intention to leave [44, 45] and organizational commitment [46]. Further, people’s values will guide their careers. De Vos et al. [47] cite the kaleidoscope career model [48], which distinguishes three values: authenticity, balance and challenge. Although all three values are always active, one value will have priority. In line with the findings by Abrahamsen [23], this may explain why registered nurses career preferences change and why primary care work becomes more popular with time. As personal needs, interests and aspirations might change, career competencies and career adaptability are important for individuals to achieve their desired career [36].

Current career literature places a significant focus on personal agency and control, but to understand a career trajectory that is becoming more complex, it is necessary to include the role of context and external events [49]. The *context* dimension refers to how the work-related context and private life affect people’s career sustainability, such as first-line managers, colleagues, patients, family, and friends. For example, numerous studies within health care have highlighted the importance of social support from immediate supervisor for registered nurses’ intention to leave the profession [50], commitment to the organization [51] and reduced intention to leave [44, 52]. The experience of conflict between work and family correlates with nurses’ choice to leave an organization and the profession [53]. Nurses will most likely experience several career shocks, defined as *disruptive and extraordinary events that are, at least to some degree, caused by factors outside the focal individual’s control and that trigger a deliberate thought process concerning one’s career* (49, p. 4). For instance, going through a divorce, having children, being diagnosed with a serious illness, accepting a new job or reorganizations are likely to affect nurses’ career choices. Changes in demands or resources at home or at work can make careers more or less sustainable over time [54, 55]. If nurses’ work becomes too demanding without an increase in the necessary resources, it could lead to stress and exhaustion according to the job demand- resource (JD-R) model [56] and affect nurses’ choice to leave home care services or the profession [57]. Emotional demands [58] and burnout [53] are associated with registered nurses’ intention to leave. Although individuals are the “owners” of their careers, both employees and employers are responsible for creating sustainable careers [59, 60]. Home care nurses’ ability to perform advanced procedures that had previously been done in hospitals has raised expectations of their work and competence [61]. To succeed and remain employable, individuals are required to manage and develop their knowledge, abilities and skills to meet changing demands [62, 63]. At the same time, employers must provide opportunities for professional learning and development [64]. Aligning one’s needs with the organization and private context, will benefit all stakeholders, and impact the sustainability of his or her career [24].

The *time* dimension relates to the dynamic evolution of careers [36]. Employees’ careers might be more or less sustainable over time due to changes in demands or resources at home or at work [54, 55]. For example, registered nurses often work part-time while the children are young [65]. Earlier research shows an age differences between younger and older nurses and their wish to leave home care services and nursing homes [66]. This is in line with previous findings of a negative relationship between registered nurses’ age and turnover intention [50, 67].

## Methods

### Setting, design, participants and ethics

This study is part of a larger research project in Leadership and Technology for Integrated Health Care Services. The project explores how home care nurses, general practitioners (GPs) and multimorbid patients experience and contribute to integrated care. In Norway, the municipalities are responsible for the organization and delivery of primary care services, and national health and regional health authorities are responsible for specialist care services. Local authorities are free to determine how to organize community services; the municipality in this study organized home care services in ten units. The responsibilities of the municipalities have increased over time and challenges have been identified in management, recruitment, competency and in the responsibilities assigned to professional groups within primary health care services [68]. A qualitative research design using individual interviews was chosen for this project, as this enabled us to have a dialogue with the study participants and explore individual experiences [69].

This qualitative study uses individual semi-structured interviews with 20 home care nurses from a medium-sized municipality in Norway (Table 1). In Norway, registered nurses have a bachelor’s degree and are authorized to practice as a nurse by the Norwegian Authority for Health Personnel [70]. The project group established contact and made a formal agreement to conduct the study with the administrative leader of the municipal division of health and social care. We used purposive sampling and approached first-line managers in relevant units by phone or e-mail, and they helped recruit registered nurses with a minimum of a bachelor’s degree and who were familiar with the patients included in the project. Potential participants received written information about the study so that they could decide whether to participate. This included information on the purpose of the project, the person in charge, what their participation involved, how data was stored and used, what would happen with the personal data at the end of the research project, rights and that participation was voluntary. The first-line managers scheduled the interviews, which were held during the participants’ working hours in a quiet room located at the nurses’ workplace. The interviews were held face-to-face with only the interviewer and participant present. None of the registered nurses have subsequently withdrawn from the research project.

**Table 1:** Characteristics of study participants

	**Age**	**Years of experience as a nurse**	**Job percentage**
Nurse 1	39	16	100
Nurse 2	49	24	100
Nurse 3	34	10	80
Nurse 4	34	11	75
Nurse 5	37	14	100
Nurse 6	43	22	100
Nurse 7	56	35	60
Nurse 8	39	11	75
Nurse 9	38	7	75
Nurse 10	63	19	100
Nurse 11	61	25	100
Nurse 12	32	10	100
Nurse 13	32	3	100
Nurse 14	53	31	75
Nurse 15	56	28	100
Nurse 16	29	1	75
Nurse 17	34	10	100
Nurse 18	33	11	100
Nurse 19	34	4	85
Nurse 20	53	30	100

The research procedures were reported to the Norwegian Centre for Research Data (ref. no. 228,630). The Regional Committee for Medical and Health Research Ethics in Norway (ref. no. 2019/1138) exempted the research project from formal review since the research project was not expected to generate new knowledge about health and disease. Before the interviews, all participants received oral information about the aim of the research project, that the interviewer was a PhD student and had the opportunity to ask questions, before signing a voluntary written consent. The study participants were informed that they could withdraw from the study at any time without consequences and could access the data collected. The participants received written contact details to the project leader, Data Protection Officer and Data Protection Services. A voice recorder was used to record the interviews. In the beginning of the interview, the participants were asked not to use any identifiable names, and the interviewers did not mention or record the name of the participants. Each participant received a study number to secure confidentiality. Anonymous transcripts and recordings were stored on a password-protected computer. A list of names and respective codes is locked in a secure cabinet at the University where the project leader is employed, and can only be accessed by the research group. In accordance with the protocol of the Norwegian Center of Research Data all collected data will be deleted in the beginning of 2025.

### Data collection

Data collection took place between October 2019 and March 2020. The semi- structured interviews ranged from 48 min to 1 h and 38 min. All were audio recorded with participants’ permission, transcribed verbatim and de-identified. One interview was incomplete, because the participant had to leave before we had asked all the questions. This interview lasted for 30 min, and we included the answers in the study. The research questions were developed by the research group, and the interview guide addressed participants’ gender, age, family situation and open-ended questions explored nurses’ thoughts, reflections, and experiences on their career in home care services and further career interests. The interviews also focused on nurses’ experience of cooperation with the patient and GP on the project. The interview guide included questions such as “How would you describe your working situation in home care services?”, “How do you envision your career as a nurse?” and “what would be important to you in terms of support/incentives/development in order to make your desired career possible?”. The researchers were not acquainted with any of the registered nurses participating in the study.

The research group consists of four females and one male. Two members of the research group are professors experienced with qualitative studies, whereas the other members are PhDs. The first author conducted 15 interviews and another member of the research group conducted five. Both interviewers were PhD students, with previous experience in conducting qualitative interviews. The first author is a female with human resources experience in specialist health care, and the other male researcher is a GP. Other members of the research group are experienced in leadership and nursing, making the group multidisciplinary. The research group had peer debriefing during the project period to discuss and gain different perspectives on the ongoing interviews. In addition, the two researchers conducting the interviews had an ongoing dialogue checking the correspondence between the findings. A sample size of 6–20 + participants is considered satisfactory in qualitative research, depending on the richness of the data and size of the project [71]. We determined that saturation had been met after about 15 interviews.

### Analysis

An inductive thematic analysis of the data was undertaken. This is a flexible way to identify themes and patterns in qualitative data analysis [72]. The analysis was guided by Braun and Clarke’s [72] six-phase process (Table 2). The main themes were generated abductively. Preexisting knowledge guided some of the interview questions, and when analyzing the data the first author read career theory to identify the relevance of information to the research aim. To address the trustworthiness of this study we applied strategies from the standardized criteria by Lincoln and Guba [73], namely credibility, transferability, dependability and confirmability. In line with Nowell et al. [74] we used these criteria as guidelines to support a rigorous thematic analysis. To ensure the trustworthiness in the first phase, all co-authors familiarized themselves with the data and individually searched for meaning and patterns enhancing the credibility of the study. The raw data and transcripts were organized in folders representing each nurse. In the second phase when generating initial codes, research triangulation enhanced confirmability. In phase three, the first author used Microsoft Excel and drew visual mind maps in the search for themes and connections. This process was documented and discussed with co-authors. In phase four and five of the thematic analysis themes were examined by co-authors and themes was reviewed in relation to the raw data before everyone agreed on the final naming. In phase six, we used the consolidated criteria for reporting qualitative studies (COREQ) as a guideline to ensure the transferability and confirmability of the research process [75].

**Table 2:** Thematic analysis as recommended by Braun and Clarke, and our analysis activity

Braun and Clarkes six phases of thematic analysis with descriptions		Our analysis activity
1. Familiarisation with the data	Transcribing data, reading and re-reading the data set, noting quotes of potential interest.	After transcription, the researchers read the interview transcripts. The first author read the interview transcripts several times, and created a time-line for each nurse to identify different career choices.
2. Systematic data coding	Systematic coding of interesting features across the data set, collecting data relevant to each code.	By the use of NVIVO 16 software system the first author worked systematically through each interview, and developed codes through this process. The codes were revised by going back and forth between interviews.
3. Generating initial themes	Comparing and collating codes into potential themes, gathering all data relevant to each theme.	The researchers interpreted and discussed the coded data to find patterns and a shared meaning that could form a theme. Mind maps and Microsoft Excel with a list of codes and data items was used in this process to discuss fit and number of themes.
4. Developing and reviewing themes	Checking themes against the coded extracts and the entire dataset.	The themes were reviewed in relation to each other, and in relation to the data set, and four themes was reduces to three themes in this process. This was a dynamic process, and there was a need to re-examine some of the activities done in phases two and three of the analysis. Further, the themes were viewed in relation to relevant theory.
5. Defining themes	Generating clear names and identify the specifics for each theme.	Naming themes was an evolving process, and several changes was made before the final version of name for each theme.
6. Write-up	Final chance for analysis, selection of appropriate extract examples and producing a report of the analysis.	The research group agreed on theme structure, the order in which to present themes and extract examples.

## Results

The data analysis produced three distinctive themes for nurses’ career choices in home care services: (1) as a result of influence from multiple stakeholders and contextual factors; (2) as a result of fit with nurses’ private life; and (3) as a result of enhancing meaning of work.

### Career choices as a result of influence from multiple stakeholders and contextual factors

The nurses described their choice to start working in home care services as resulting from coincidences, stakeholder influence, organizational policies and educational factors. Some participants started working in a part-time position in home care services while they were in their late teens. One nurse said:

I started working here as a student during my second year. Then a family member of a friend asked if I would like to work here as an extra and I thought yes, I could try that. And I’ve been here ever since. So yeah, it was really just coincidental that I ended up working in home care services. (Informant 18)

Clinical practice placements, part of the bachelor program in nursing, take place in hospitals, nursing homes, and home care services. The hospitals and municipalities are responsible for organizing the clinical practice, where a student gains experience with departments under clinical supervision. One nurse said:

We have many practicums at school and for my last one I also chose home care services. It was really just because I thought it had been the most fun practicum, and I ended up here. (Informant 16)

Clinical practice placements or part-time jobs familiarize nursing students with home care services as a potential employer. A good work environment, supportive colleagues and first-line managers, interesting work tasks and autonomy were among the factors influencing some nurses’ view of home care services as a potential workplace. Other nurses applied for a position in home care services as a result of changes in their personal life, like moving to a new city. We also found that different stakeholders and organizational factors influenced nurses’ choice to apply for postgraduate education or become a resource nurse. Nurses emphasized the importance of financial support from the employer in entering a specialization. The municipality provides financial support for unpaid leave and school expenses for relevant specializations. People or experiences in nurses’ surroundings often influenced their choice of specialization. One nurse explained how she had been inspired by the skilled geriatric nurses she met during clinical practice. Another nurse described why she became a resource nurse in palliative care:

It was basically because they asked me. They probably thought it was a good fit for me, even though I didn’t think so myself at that time because I thought it was a bit scary to speak to people who were in their last stage of life. “I don’t think I would be good at that” I said, but then I thought well, I just have to give it a go. So that’s what happened. I don’t really know another reason. (Informant 8)

Especially first-line managers appears to have both a positive and a negative effect on nurses’ career choices in home care services. The nurses had different experiences of management. Some unit leaders were inspiring, encouraging and supportive, while other units experienced instability and absence of a first-line manager. One nurse had applied for unpaid leave, to start working in a nursing home. Her unit had been chronically understaffed. She thought that her managers were not advertising vacancies, and were inattentive to employees’ needs. When she read a newspaper article reporting that politicians would not provide more resources to home care services, she doubted that she would return. She said:

That’s the reason I feel that I can’t do this anymore. I feel that I give, give, give all the time, while my superior is away a lot, and that really affects my motivation. (Informant 17).

### Career choices as a result of fit with nurses’ private life

An overarching theme that explains nurses’ career choices in home care services can be seen as a result of fit with their private life. It captures the ways in which nurses make career choices that improve their work-life balance and how their needs change. Some of the nurses had previously worked, or considered working, at a hospital in a nearby municipality. However, the geographical location of work, shift arrangements and family situation affected nurses’ choice to work in home care services. As one nurse said:

From 2013, I think it was, when I started working a bit at the hospital and I thought I should give it a go again. So I was there a couple of years, I think, but then it became quite hard to combine with family life, especially because my husband travels a lot. That just made it too hard to work there. (informant 6).

Some nurses applied for a position in home care services, as the workplace is closer to home, and they would have a shorter commute and spend less time in traffic. Even though work in a hospital is considered professionally attractive, some nurses found it difficult to combine with their family life. As the example illustrates, when both parents have irregular work schedules, organizing family life is not easy. This was especially true for nurses with children, who struggled to balance their responsibilities to work and family. One nurse said:

After I had children it’s been quite practical. I didn’t have to work a three split schedule for example. My evening shifts start 3.30 pm or 4.30 pm. It’s a flexible job, and because you start at 7.30 am you are able to bring your children to nursery first. (Informant 18)

Nurses identified working hours to be better in home care services than at a hospital or nursing home. Shift arrangements in home care services involves more flexible working hours and does not include work at night. However, nurses still experience shift work as demanding, as it includes evening, weekend, and holiday work. To accommodate this kind of schedule, nurses had to depend on a partner who worked standard business hours and who could pick up the slack with family responsibilities. Nurses described how they and their partner shared household and childcare responsibilities. Several nurses who had small children worked fewer hours to spend more time at home. However, none of the nurses mentioned having a male partner taking unpaid leave to be at home with their children, illustrating a traditional gendered division of childcare. A nurse said:

When the children were little we really had enough just trying to keep our heads above water so I worked part time and as the children have had less need of me I’ve increased my work hours. I’ve felt that my work at home has been the most important one, and that I’ve worked as a nurse in addition to that one. But as time has gone by, and I’ve gotten more energetic, and my children get by more on their own, I’ve been working full time. (Informant 20).

In Norway, parents are entitled to 12 months of paid leave. In addition, each parent is then entitled to one year of unpaid leave. Most children between the ages of one and five attend kindergarten. There is one admission every year facilitated by the municipality. This means that some parents need to apply for unpaid leave so that they can stay home with their children who are waiting to start kindergarten. Several nurses chose to work part-time evening shifts while waiting for a place in kindergarten, so they were able to combine work and family. Other nurses whose children were eligible for kindergarten preferred to apply for unpaid leave in order to stay at home with them. Their colleagues and first-line manager supported their decision to work part time and adjust their working situation. However, as their children became less dependent on them, some nurses opted to return to work full time, in the evenings, and apply for a specialization. Nurses felt a tension between personal and professional wishes. One nurse described how she wanted to take a specialization, but adjusted the time and place to accommodate her children. However, younger informants did not want to delay pursing a specialization or master’s degree for too long. Nurses in the later stages of the careers and without a specialization, supported this view, as they believed that they were too old for further education. However, they expressed an interest in developing their skills and knowledge at work.

### Career choices as a result of enhancing meaning of work

Nurses can work in different clinical fields and types of organizations. Work content and organization of work influenced participants’ application for a position in home care services. One nurse explained why she started in home care services:

It’s very special to go into people’s houses, it’s a very pleasant atmosphere. You get to see the whole person in a way, not just their illnesses. And you get to see how they live, which gives you an idea of who they are as people. It’s also very exciting to hear their stories and not just see them when they are at their lowest. (Informant 5).

In home care services, many patients receive treatment for years, so their nurses know their complete history, needs, routines, and interests, all of which affect quality of care. Nurses can take a holistic approach to patients. An important part of nurse’s job is to monitor changes in a patient’s condition. Spending time in the patient’s home and getting to know them and their family help nurses to understand that patient’s needs. A nurse described why knowing the patients is important:

I think it’s important. Because you care about their well-being, and….The fact that you can get a bit close to them so that you’re able to help in the best possible way. And to not just see their illnesses, but also everything around them. Their next of kin, contact with their doctor….to be able follow up properly. (Informant 13).

Knowing patients well is an antecedent for providing quality of care, something the organization of work in home care services facilitate. Nurses enjoyed the coordination of care among stakeholders, like the patient’s family, GP, physiotherapy services and allocation office. However, nurses were frustrated with the lack of collaboration with GPs or hospitals, because it led to uncertainty and extra work for nurses who often work alone in patient’s homes. Although it can be difficult to work independently, it can also be motivating. One nurse said:

Yes, I did consider the hospital. I thought it might be more challenging, as there are a lot of procedures. But the thing with home care services is that you work quite independently because you’re out there driving. So I figured I’m learning just as much here, and maybe even more. You become independent, and you have to make your own choices and I feel more in charge of my own work situation here. (Informant 14).

The nurse mentions the importance of having an interesting job and recognizes autonomy as a factor in her choice to work in home care services. When driving from patient to patient, nurses have time to reflect. In addition, informants expressed happiness at not being tied to an institution. At the same time, they noted the importance of professional support, and described daily arenas where they were able to discuss challenges and patients’ conditions with colleagues and first-line manager. They also discussed patient’s conditions with the patients themselves, their families, GP, and contacted acute care if necessary. The motivation for enhancing the welfare of others influenced nurses’ career choices, like specialization, becoming a resource nurse and leaving home care services. Several of the informants had taken a specialization. According to one nurse:

And that’s why I wanted to do further postgraduate studies too. I felt I needed it. And I feel that it’s good that we are three, rotating it, because there are so many wounds it’s needed. (Informant 5).

Nurses described how work in home care services has become more specialized in the past decade, and diagnoses have become more complicated. Hospitals discharge patients sooner and nurses are expected to perform unfamiliar procedures. To provide quality of care, nurses stressed the importance of professional knowledge. However, some considered postgraduate education as a possible alternative to home care services and shift work. Some nurses who study for a master’s degree were unsure about their future in home care services, and how the municipality would make use of their competence after graduation. One nurse with a specialization had resigned from her job in home care services to accept a position in the specialist health care services. Work in home care services is diverse, as nurses usually serve a variety of patients with different diagnoses. She had thrived in home care services, but wanted to use her skills to help patients with more serious diagnoses.

## Discussion

The aim of this study was to increase our knowledge of home care nurses’ career choices. Three themes emerged: (1) career choices as a result of influence from multiple stakeholders and contextual factors; (2) career choices as a result of fit with nurses private life; and (3) career choices as a result of enhancing the meaning of work. Based on the sustainable career perspective [24], we expected that the dimensions of person, context and time would relate to the career choices of home care nurses. Previous career literature has been criticized for putting too much attention on people’s agency [14, 49, 76]. This study advances knowledge by highlighting the importance of context and time on registered nurses’ career choices, and provide support for the use of the sustainable career perspective as a broad theoretical framework in understanding registered nurses career choices. It contributes to a field where previous research is largely based on quantitative data [17, 23, 31] and illustrates how nurses themselves, their private context and work context influence their career choices over time.

The results identified how stakeholders and factors within multiple contexts influenced nurses’ career agency over time. Clinical practice and the offer of a part-time job, considered a positive career shock, provided job resources and experience. This appeared to affect nurses’ perception of home care services as a potential employer, the person-job fit, and their choice to apply for a permanent position after graduation. This supports previous research [18, 77, 78], in which clinical experience and curriculum content are identified as the main tools for changing nurse students’ negative perceptions about work in primary health care [79]. The implication of this is that managers and employees in home care services are proactive and encourage people to work in home care services and create opportunities for learning and development in line with registered nurses and home care services needs for competence. This will benefit both employer and employees need for development [80]. Home care services could offer mentors, role models, interesting work tasks, encourage voice, feedback and support in order to provide high-quality work experiences, as lack of support, uninspiring work tasks, and time constraints could lead to stress and frustration and preclude employment in home care services [25, 27, 81].

Two nurses had applied for a position outside home care services; however, the motives and types of agency behind their choices differed. One nurse wanted to quit because of increased workload, time pressure, and lack of support from managers and politicians (push factors), resulting in an unsustainable career. The other nurse was drawn to another job where her competence would be put to better use (pull factors). Previous research has identified burnout as a threat to career sustainability by causing career turnover [42]. Time pressure and heavy workload may hinder nurses from performing work that meets their professional standards, leading to stress and frustration. In line with the JD-R theory [56], high demands and low resources over time can decrease person-job fit, which causes nurses to leave home care services for a more sustainable career. However, research has indicated that employers can mitigate the negative effects of increased work demands by offering job resources [54]. In line with previous research [29, 44, 50], this study highlights the important role of first-line managers for nurses’ career choices. To develop first-line managers skills by offering training programs which focus on understanding employee needs, how to provide support and encourage nurses career development will be important. This can prevent nurses from seeking other job opportunities, something that will serve the interests of home care services by ensuring a stable workforce.

Nurses started to work and continue to work in home care services as it fits their private life, supporting previous research identifying work-life balance as one of the most satisfying aspects of work in primary health care [3, 25, 82]. When nurses become mothers, they seem to give the top priority to balancing work and home. Our results show that organizational factors such as the location of work, shift arrangements, and the possibility to adjust work based on the demands of family life becomes important for nurses’ choices. Previous research has showed that working night shifts is associated with negative family outcomes such at work-family conflict, especially when children are small [83]. Nurses adapt to family demands by working part-time, changing their hours of work and postponing their plans for higher education. However, none of the nurses stated that their choices depended on the limits set by a full-time working partner. This study supports the importance of including non-work domains in research on sustainable careers [82, 84]. Kossek and Ollier-Malaterre [59] and Straub et al., [60] have emphasized the importance of both employees and employer to foster sustainable careers. This implies that to retain registered nurses, home care services should adjust HR politics and practices with employees’ expectations, norms and values through different phases of life, as this can facilitate nurses’ experience of fit between their personal life and their work in home care services. For several nurses the desire to work part-time appears to be temporary for parts of their lives when they experience increased family obligations, supporting earlier findings [20]. Registered nurses in the last phase of their career seem to have other career aspirations than nurses in their first phase. As the proportion of adults is increasing, health care organizations should motivate registered nurses to continue until a later age. In line with suggestions by Kooij et al. [85], municipalities can offer HRM practices such as training, career planning or lateral job moves.

The results show that nurses experience meaning of work by helping their patients. Autonomy, helping people and having a close relationship with patients have been identified as some of the most satisfying aspects of work in primary health care [3, 25]. The ability to derive meaning from work is important for people’s psychological well-being [86], and this highlights the importance of considering meaning of work as an important resource. Some nurses proactively shaped their careers by undergoing or completing postgraduate education. In line with self- determination theory [39], nurses expressed a need for knowledge, to improve patient care for a growing number of patients with complicated diagnoses. Studies indicate that home care nurses must perform increasingly advanced procedures and assessments, and call for more information and training about specific procedures [8]. To encourage, facilitate, and support registered nurses’ development of competence will be important. In line with previous research [6, 87], our findings demonstrate the importance of home care services continuously working to ensure improved collaboration with other health care providers in order to reduce uncertainty and extra work for registered nurses. A sense of accomplishment has been identified as important for nurses’ intention to remain in home care services [28], and our results indicate that this guides additional career choices. In line with the principle of conservation of resources [38], acquiring resources makes nurses more employable and provides them with career opportunities inside and outside primary health care. Some nurses who studied for a specialization were unsure about their future in home care services, and did not see their employer as taking the initiative in discussing possible career paths. Providing career planning support, with a perspective of possible career alternatives within home care services that are valuable to the organization and provides meaning to registered nurses will be important to develop and retain registered nurses. At the same time, nurses need to be aware of what matters to them and act in the interests of their own needs and values. This will improve the chance of person-career fit and of a sustainable career [36]. Home care services should align work with nurses’ interests, strengths, and values, as this would benefit both the municipality and nurses in terms of improved job performance, meaningfulness, and organizational commitment [64].

## Limitations

This study has several limitations. First, the sample consisted only of women from a single municipality in Norway. Further research should be conducted in different health care settings and cultures. A second limitation is that the results may be biased as it can be hard to recall what happened many years ago. Additional research should use a longitudinal design to increase our understanding of nurses’ career choices. Finally, future research should examine the role of age and the perspective of the organization.

## Conclusion

The aim of this study was to increase our understanding of nurses’ career choices to offer insights that can be used to attract, motivate, develop and retain registered nurses in home care services. The results illustrate the importance of having a whole life perspective to understand nurses’ career choices, and how nurses’ career preferences change over time. To meet the population’s increased need for health and care services it is important for the municipality to facilitate sustainable careers across the life span through HR policies, motivating and stable managers, which support nurses changing needs, interests and values. Nurses need to be mindful and act according to what is most important to them.

## Data Availability

The datasets generated from the study are not publicly available due to reasons of confidentiality. Additional knowledge of the de-identified data can be available from the corresponding author on reasonable request.

## Declarations

### Authors’ information

GHH, MSc, HR Adviser at Stavanger University Hospital and currently a PhD candidate at Stavanger University Hospital and University of Stavanger Business School. OØ, PhD, Research associate professor at NORCE Norwegian Research Centre. MS, PhD, Professor at the Faculty of Health Sciences, University of Stavanger, AM, PhD, Professor at the University of Stavanger Business School.

## List of abbreviations

GPs: General practitioners
